# Evaluating the performance of an automated respiratory rate counter in detecting fast breathing pneumonia in children using a reference video expert panel

**DOI:** 10.1186/s44247-025-00175-3

**Published:** 2025-08-21

**Authors:** Ahad Mahmud Khan, Md Shafiqul Islam, Nabidul Haque Chowdhury, Salahuddin Ahmed, Rezwana Tabassum, Sadia Afrin, Zannatul Ferdush Amin, Kazi Sazzadul Haque, Afroza Yeasmin Rumi, Jawata Rahman, Rakib Bhuiyan, Rizouan Ur Rashid, Kamrun Nahar, Robynne Simpson, Ayaz Ahmed, Md Mozibur Rahman, Ting Shi, Abdullah H. Baqui, Steve Cunningham, Eric D. McCollum, Harry Campbell

**Affiliations:** 1 Projahnmo Research Foundation, Dhaka, Bangladesh; 2 Usher Institute, University of Edinburgh, Edinburgh, UK; 3 University of Greenwich, London, UK; 4 Department of Paediatrics, Shaheed Suhrawardi Medical College Hospital, Dhaka, Bangladesh; 5 Royal Hospital for Children, Glasgow, UK; 6 Department of Neonatology, Institute of Child and Mother Health, Dhaka, Bangladesh; 7 Department of International Health, Johns Hopkins Bloomberg School of Public Health, Baltimore, MD USA; 8 Centre for Inflammation Research, University of Edinburgh, Edinburgh, UK; 9 Department of Paediatrics, School of Medicine, Global Program in Paediatric Respiratory Sciences, Eudowood Division of Paediatric Respiratory Sciences, Johns Hopkins University, Baltimore, MD USA

**Keywords:** Pneumonia, Respiratory rate, Fast breathing, Automated counter, Video expert panel, Reference standard

## Abstract

**Background:**

According to the World Health Organization’s Integrated Management of Childhood Illness (IMCI) guidelines, childhood pneumonia diagnosis relies on counting respiratory rate (RR). Counting RR by health workers is frequently inaccurate, leading to misdiagnosis and poor outcomes. Automated RR counters could potentially overcome these limitations. To address this gap, we introduced an automated RR counter and developed a reference video expert panel (VEP) to evaluate its performance.

**Methods:**

We conducted a cross-sectional study involving children aged 0–59 months with suspected pneumonia in Bangladesh. The RR of children was counted using an automated counter (ChARM) and chest movements were simultaneously videotaped. These videos were interpreted by the VEP, trained to a standard procedure. We assessed ChARM’s accuracy in comparison to the RR generated by the VEP and summarised the time taken to count RR by ChARM.

**Results:**

Among 339 enrolled children, ChARM successfully counted the RR of 294 children (86.7%). The VEP reached a consensus (i.e., RR count difference within two breaths per minute (bpm) between two VEP members) in 257 of the 294 children (87.4%). ChARM and the VEP agreed on RR counts within two bpm in 68.1% of children (*n* = 175/257), with a mean difference of 1.7 bpm and limits of agreement ranging from -6.7 to 10.2 bpm. ChARM classified age-adjusted fast and normal breathing with a sensitivity of 95.8% (95% CI: 88.1–99.1) and a specificity of 93.5% (95% CI: 89.0–96.6), demonstrating high agreement (kappa = 0.86). The median time to count the RR by ChARM was 66 s (interquartile range: 61–73 s).

**Conclusions:**

ChARM counted RR accurately against a VEP reference, indicating a potential role in supporting health workers to diagnose pneumonia. However, it was unsuccessful for 1 in 8 cases, typically those more clinically challenging, suggesting a possible systematic bias. Further research is needed to address these issues and confirm ChARM’s reliability for broader use in real-world settings.

**Trial registration:**

Current Controlled Trials ISRCTN14120515, registered retrospectively on 19 September 2024.

**Supplementary Information:**

The online version contains supplementary material available at 10.1186/s44247-025-00175-3.

## Introduction

Pneumonia is one of the leading causes of under-five mortality worldwide [1]. In 2015, there were 138 million episodes of pneumonia and 0·9 million deaths attributed to it [2]. In low- and middle-income countries (LMICs), about 101.8 million pneumonia episodes were estimated in under-five children in 2015, with an incidence of 0.15 episodes per child-year [3]. Pneumonia is the number one infectious killer of children, accounting for about 17% of all deaths in Bangladesh [4].

According to the World Health Organization (WHO) Integrated Management of Childhood Illness (IMCI) guidelines, a crucial step in diagnosing pneumonia involves measuring a child’s respiratory rate (RR) by observing chest movements and counting RR manually for 60 s to identify age-specific fast breathing [5]. Despite being a widely used method, manual RR counting has several drawbacks. It depends on the observer’s capability to count RR accurately, making it prone to errors. In busy healthcare settings, where healthcare providers have multiple responsibilities, manual RR counting may not be feasible and is often neglected [6, 7]. It is also challenging to count RR by observing the chest movements while using a timer or watch, and some may struggle to remember the count [8]. This often leads to misclassification of fast breathing, resulting in incorrect pneumonia diagnoses and inappropriate treatment [9].

Automated RR counters are primarily used for continuous monitoring of RR in high-resource settings, and these tools are typically highly technical [8, 10]. This improved technology for counting RR has not yet been widely adopted in low-resource settings. The availability of improved diagnostic tools to support frontline health workers might improve pneumonia diagnosis. A few automated RR counting devices have been developed for use by frontline health workers, including the Children’s Automated Respiration Monitor (ChARM) [11, 12], Rad-G [13, 14], uPM60 [15], and Respimometer [16].

ChARM converts chest movements detected by accelerometers into a precise breathing count using specially designed algorithms. The device is strapped around the child’s abdomen, automatically counts RR, and classifies fast breathing [11, 12]. The manufacturer claims the device has an acceptable precision level of ± 2 breaths per minute (bpm) when measured under recommended conditions [17]. Its usability and acceptability have been tested in studies conducted in Ethiopia [18] and Nepal [19]. From the perspective of clinical importance, applying the device is not considered to significantly affect the RR of children immediately after attachment [20].

Currently, there is no universally recognised gold standard reference for evaluating the accuracy of automated RR counters [21]. Most existing studies have used a trained and standardized medical professional’s (e.g., physician, nurse) manual RR count as the reference standard, though manual RR counting is considered unreliable [8]. Possible biases using human expert counts as the reference standard include difficulty in measuring the RR over the same simultaneous period and inconsistencies in human RR counting [21, 22].

Videography of a child’s chest movements and interpretation by several experts – referred to as a video expert panel (VEP) – could serve as a reference standard, if standardised [21, 22]. This method has been implemented to evaluate health workers’  accuracy in manually counting RR in a previously published study [23].

Novel diagnostic aids might help health workers improve childhood pneumonia diagnosis. Evaluating these devices before implementing them at the field level is essential to ensure accurate readings. Factors such as the time required to count RR and the potential impact of the device on the RR are also crucial for successful implementation of a new technology. The ChARM device is specifically designed to identify fast-breathing pneumonia in low-resource settings. However, its performance has not been widely explored in the literature. Therefore, this study was designed to evaluate the performance of the ChARM device in measuring RR compared to the VEP reference RR count.

## Methods

### Study design and setting

This study was conducted in accordance with the Consolidated Standards of Reporting Trials (CONSORT) guidelines. The detailed methodology has been published previously [24]. This cross-sectional study was conducted between 2021 and 2022. We recorded videos at different levels of health facilities in Bangladesh, including three community clinics (CCs) and Zakiganj Upazila Health Complex (UHC – a sub-district hospital) in Sylhet, and the Institute of Child and Mother Health (ICMH) in Dhaka. CCs, which are staffed by community healthcare providers (CHCPs), represent the lowest tier of healthcare facilities in Bangladesh [25]. We trained and standardized six physicians and developed the VEP, which served as the reference standard for evaluating the RR counter.

### Study population

The study population included infants under two months of age presenting with any illness, and children aged 2–59 months presenting with cough and/or difficulty breathing. We excluded children who presented with any danger sign, as well as those whose parents refused to provide consent.

### Sample size and sampling method

Bland–Altman’s statistical method was used to calculate the sample size [26, 27]. Assuming a type-I error rate (α) of 0.05, an expected mean difference of 0.5 bpm, an expected standard deviation of 1.5 bpm, and limits of agreement of 4 bpm, a minimum sample size of 226 children was required to achieve 90% power to assess agreement in RR counts between ChARM and the VEP. A total of 339 children were consecutively enrolled in the study.

### Training procedure for VEP

Six local physicians holding Bachelor of Medicine and Bachelor of Surgery (MBBS) degrees underwent training to assess RR using an online platform using videos with pre-determined RR counts. The training adhered to the WHO IMCI guidelines [28, 29]. Upon completing the training, each physician was assigned 20 videos with different levels of breathing. The accuracy of their RR assessments was determined based on whether their RR counts fell within ± 2 bpm of the pre-determined values. All six physicians met the passing criteria and were deemed qualified to serve as members of the VEP.

### Study procedure

The Canon EOS M50 camera [30] was used to record videos of children’s chest movements. The physician or CHCP attached the ChARM device, and the research staff started recording. Once the child was calm, the physician or CHCP switched on the ChARM device, and the video recording continued until the device finished its count. The time taken by the ChARM device to obtain the RR was noted. A maximum of three minutes was permitted for the device to register a reading or display an error message. If neither occurred, it was recorded as a failed attempt. The physician or CHCP was allowed up to three attempts for each reading.

The videos were transferred to a password-protected computer. After necessary editing, each video was assigned to two randomly selected VEP members for RR interpretation. If there was a consensus that a video was uninterpretable, it was excluded. If the difference in RR between two members was ≤ 2 bpm, their average RR was considered as the final RR. If there was a disagreement in interpretability or the RR difference was > 2 bpm, the video was sent to a third member. If discrepancies persisted among all three panel members (i.e., no two members had a RR difference of ≤ 2 bpm), the video was reviewed by a fourth member. If the fourth member agreed with any previous member, the average RR of the two was considered final. If no agreement was reached among any two members, the video was excluded from the analysis (Supplementary figure 1).

### Operational definitions

#### Concordant interpretation

The concordant interpretation was defined as:

- ▪ Both VEP members interpreting the video as uninterpretable, or
- ▪ Both interpreting it as interpretable with a RR difference of ≤ 2 bpm.

#### Discordant interpretation

Discordant interpretation was defined as:

- ▪ One VEP member interpreting the video as interpretable and other as uninterpretable, or
- ▪ An RR difference of > 2 bpm between the two members.

### Statistical analysis

A Bland–Altman plot was created to show the agreement between ChARM RR counts and VEP counts. The percent agreement within ± 2, ± 3, ± 4 and ± 5 bpm between ChARM and VEP RR was calculated. Sensitivity, specificity, positive predictive value (PPV), and negative predictive value (NPV) for identifying fast breathing were computed considering the VEP as the gold standard. Cohen’s kappa statistic was also calculated. To evaluate the time required by the ChARM device to count the RR, the mean, standard deviation (SD), median, and inter-quartile range (IQR) were reported. Multiple linear regression was used to determine the factors associated with prolonged RR counting time. Variables with a *p*-value < 0.2 in the bivariable analysis were included in the multivariable model.

## Results

**Fig. 1:**
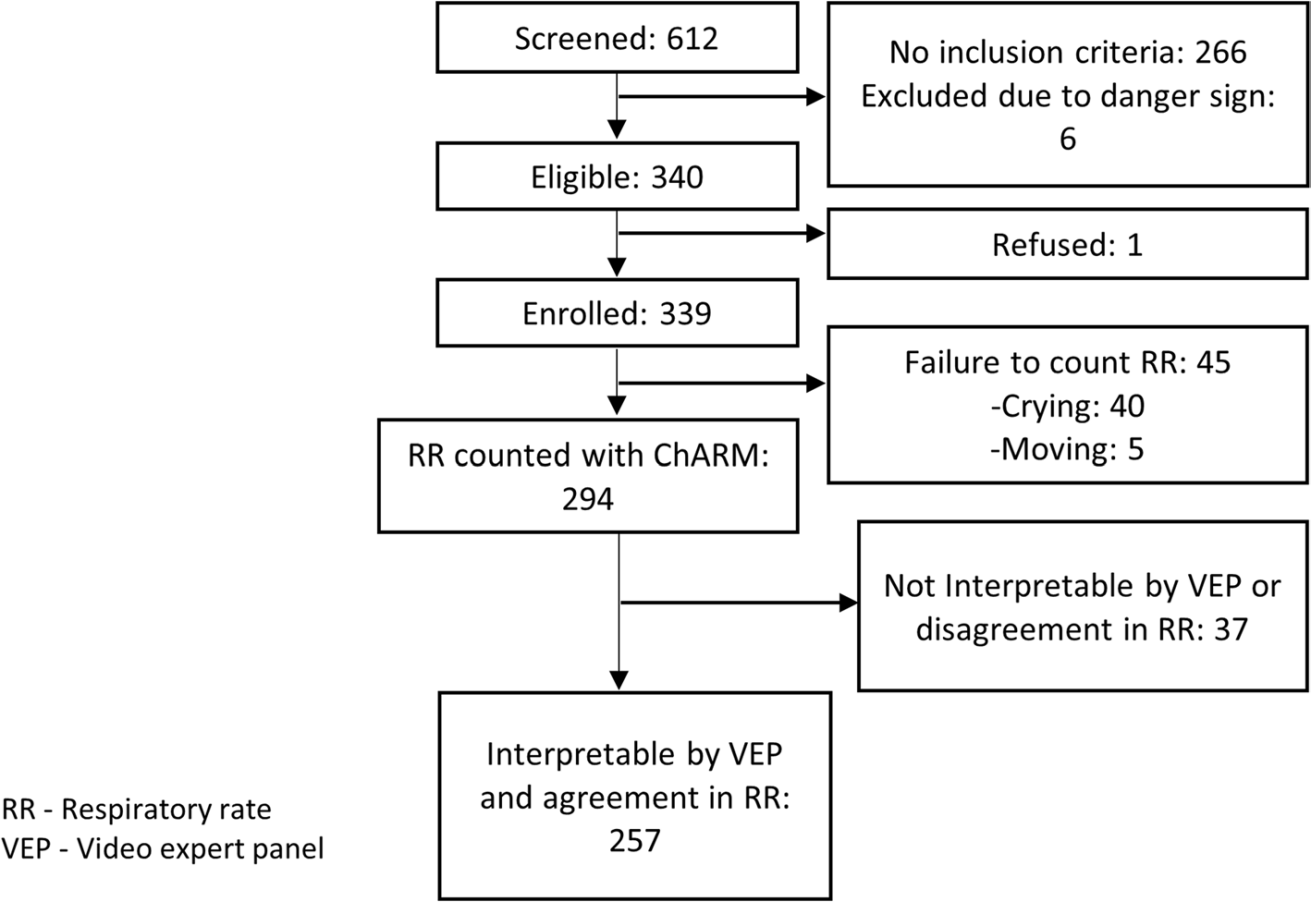
CONSORT diagram

Of the 612 children screened, 339 were enrolled, and the RR of 294 children was successfully counted automatically using ChARM. Among them, the VEP successfully counted the RR of 257 children, who were included in the final analysis (Fig. 1).

### Basic characteristics of the children

Table 1 presents the basic characteristics of the children. The median age was 13 months (IQR: 5–36 months), with a male-to-female ratio of 1.4:1. The most common condition during assessment was calm (47.5%), followed by asleep (42.8%). The RR of more than half of the children (59.9%) was below 40 bpm.

**Table 1:** Basic characteristics of the children

Characteristics	Number (%)
Age (months)	
0–2	25 (9.7)
2–11	94 (36.6)
12–35	66 (25.7)
36–59	72 (28.0)
Mean (SD)	20.8 (18.7)
Median (IQR)	13.0 (5.0–36.0)
Sex	
Male	150 (58.4)
Female	107 (41.6)
Health facility	
ICMH	80 (31.1)
UHC	80 (31.1)
CC	97 (37.7)
Child condition during assessment	
Calm	122 (47.5)
Asleep	110 (42.8)
Some movements	23 (8.9)
Crying	2 (0.8)
RR interpreted by VEP (bpm)	
< 40	154 (59.9)
40–59	76 (29.6)
≥ 60	27 (10.5)
Fast breathing interpreted by VEP	
Yes	71 (27.6)
No	186 (72.4)
Total	257 (100.0)

*bpm* breaths per minute, *CC* Community clinic, *CHCP* Community Health Care Provider, *ICMH* Institute of Child and Mother Health, *IQR* Inter-quartile range, *RR* Respiratory rate, *SD* Standard deviation, *UHC* Upazila Health Complex, *VEP* Video expert panel

### Agreement in respiratory rate count between ChARM automated count and video expert panel count

**Fig. 2:**
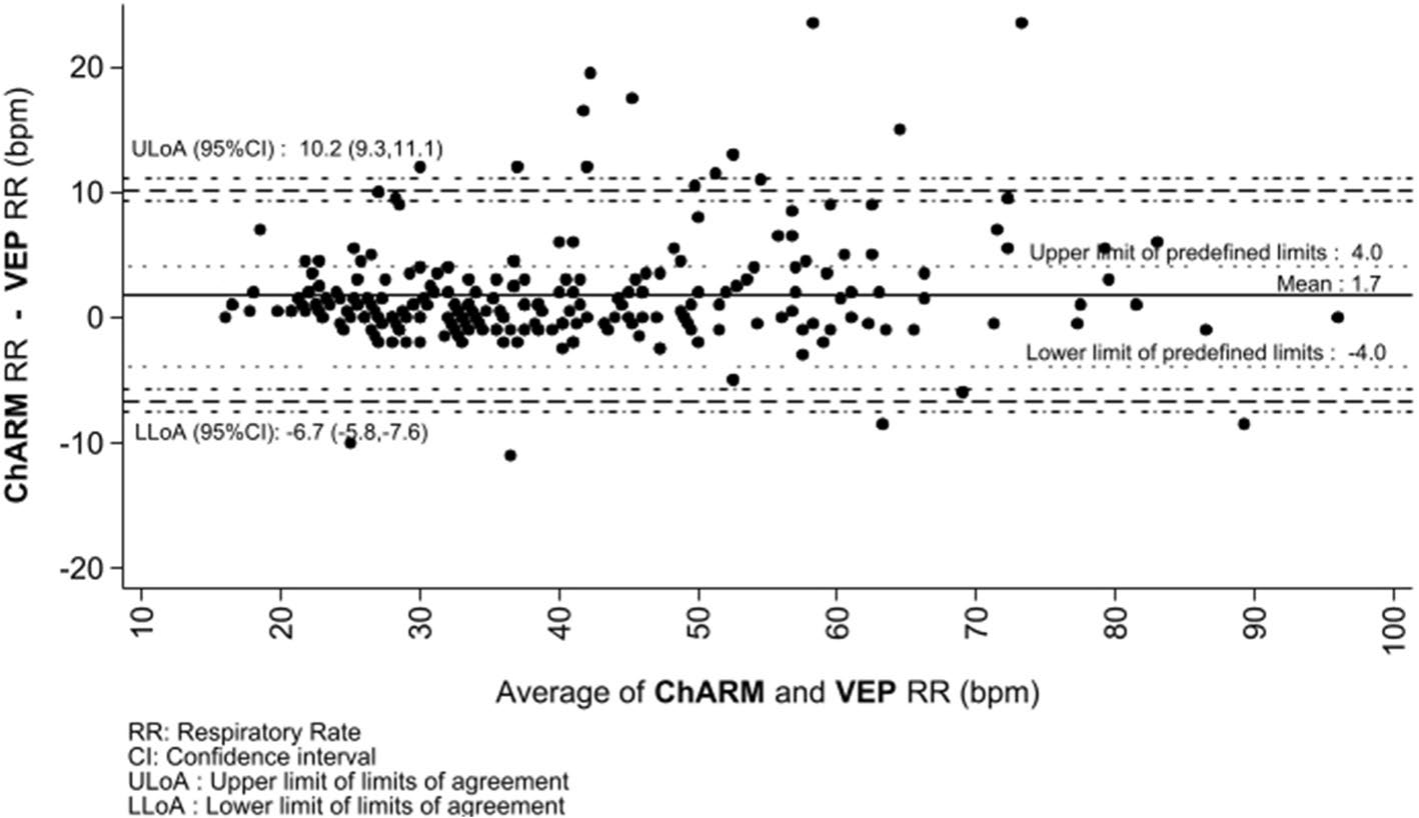
Bland Altman plot showing the agreement between respiratory rate count by the ChARM and respiratory rate count by video expert panel

The Bland–Altman plot illustrates the agreement between the ChARM RR count and the VEP count (Fig. 2, Supplementary figure 2). The mean difference in RR counts was 1.7 bpm, indicating that ChARM tended to produce slightly higher RR than the RR counted by the VEP. Most of the data points concentrated toward the mean difference line, suggesting that a good agreement between the two methods for most children. However, a few outliers with large differences were observed, indicating a certain amount of inconsistency. The limits of agreement ranged from −6.7 to 10.2 bpm. This wide range demonstrated high variability in differences between the two measurements.

Table 2 presents the proportion of RR agreements between ChARM and VEP within specified bpm ranges. Overall, the agreement was 68.1%, 76.7%, 81.3% and 85.6% within ± 2 bpm, ± 3 bpm, ± 4 bpm and ± 5 bpm, respectively. The agreement was lower among children below two months of age, female children, those assessed at the UHC, children who were moving or crying, those with higher RR, and those classified as having fast breathing. The agreement was found associated with health facility (*p* < 0.05), child condition (*p* < 0.01), RR count (*p* < 0.01), and presence of fast breathing (*p* < 0.05).

**Table 2:** Agreement in respiratory rate count between ChARM and video expert panel

Characteristics	Total number	Agreement in RR count			
		Within ± 2 bpm, N (%)	Within ± 3 bpm, N (%)	Within ± 4 bpm, N (%)	Within ± 5 bpm, N (%)
Age (months)					
0–2	25	13 (52.0)	18 (72.0)	18 (72.0)	20 (80.0)
2–11	94	62 (66.0)	67 (71.3)	72 (76.6)	75 (79.8)
12–35	66	49 (74.2)	56 (84.8)	58 (87.9)	60 (90.9)
36–59	72	51 (70.8)	56 (77.8)	61 (84.7)	65 (90.3)
Sex					
Male	150	107 (71.3)	115 (76.7)	122 (81.3)	127 (84.7)
Female	107	68 (63.6)	82 (76.6)	87 (81.3)	93 (86.9)
Health facility					
ICMH	80	58 (72.5)	62 (77.5)	63 (78.8)	66 (82.5)
UHC	80	38 (47.5)	46 (57.5)	56 (70.0)	63 (78.8)
CC	97	79 (81.4)	89 (91.8)	90 (92.8)	91 (93.8)
Child condition					
Calm	122	80 (65.6)	94 (77.0)	102 (83.6)	108 (88.5)
Asleep	110	90 (81.8)	95 (86.4)	97 (88.2)	101 (91.8)
Some movements	23	4 (17.4)	7 (30.4)	9 (39.1)	10 (43.5)
Crying	2	1 (50.0)	1 (50.0)	1 (50.0)	1 (50.0)
Assessing healthcare staff					
Physician	160	96 (60.0)	108 (67.5)	119 (74.4)	129 (80.6)
CHCP	97	79 (81.4)	89 (91.8)	90 (92.8)	91 (93.8)
RR interpreted by VEP (bpm)					
< 40	154	116 (75.3)	128 (83.1)	154 (87.0)	140 (90.9)
40–59	76	44 (57.9)	53 (69.7)	58 (76.3)	62 (81.6)
≥ 60	27	15 (55.6)	16 (59.3)	17 (63.0)	18 (66.7)
Fast breathing					
Yes	71	40 (56.3)	45 (63.4)	50 (70.4)	54 (76.1)
No	186	135 (72.6)	152 (81.7)	159 (85.5)	166 (89.2)
Total	257	175 (68.1)	197 (76.7)	209 (81.3)	220 (85.6)

*bpm* breaths per minute, *CC* Community clinic, *ICMH* Institute of Child and Mother Health, *UHC* Upazila Health Complex, *RR* Respiratory rate, *VEP* Video expert panel

### Accuracy of ChARM in classifying fast breathing

Table 3 shows the accuracy of the ChARM classification of fast breathing, considering VEP classification as the reference standard. About 31.1% of children (80/257) were classified as having fast breathing by ChARM, compared to 27.6% (71/257) by the VEP. According to the kappa-statistic of 0.86, the overall categorical agreement was substantial. The diagnostic accuracy of the ChARM in identifying fast breathing compared to VEP, and the kappa values, were consistent in terms of age, sex, and health facility. However, the agreement was moderate (kappa = 0.55) when the child had some movements during the assessment (Supplementary Table 1).

**Table 3:** ChARM classification of fast and normal breathing compared to video expert panel classification

ChARM classification	VEP classification		
	Fast breathing	Normal breathing	Total
Fast breathing	68	12	80
Normal breathing	3	174	177
Total	71	186	257
Agreement (95% CI)	94.2% (90.6–96.7)		
Kappa	0.860		
Sensitivity (95% CI)	95.8% (88.1–99.1)		
Specificity (95% CI)	93.5% (89.0–96.6)		
PPV (95% CI)	85.0% (75.2–92.0)		
NPV (95% CI)	98.3% (95.1–99.6)		

*ChARM* Children’s Automated Respiration Monitor, *CI* Confidence interval, *NPV* Negative predictive value, *PPV* Positive predictive value, *VEP* Video expert panel

### Time to count respiratory rate

Table 4 describes the time taken by the ChARM to count RR according to child age, sex, health facility, child condition, and RR. The multiple linear regression model presented in Table 5 shows that the child’s condition and RR were significantly associated with the time required by ChARM to generate an automated RR count. The beta coefficients were as follows: moving or crying child (β = 7.7, p = 0.002), RR of 40–59 bpm (β = −4.5, *p* = 0.009), and RR ≥ 60 bpm (β = −7.1, *p* = 0.008). This suggests that children who were moving or crying and those with lower RR required more time for the ChARM to complete the RR measurement.

**Table 4:** Time to count respiratory rate by ChARM

Characteristics	Number	Time to count RR (seconds)		***p***-value
		Mean (SD)	Median (IQR)	
Age (month)				
0–2	25	68.1 (9.9)	67.0 (60.0–72.0)	< 0.001
2–11	94	65.6 (9.3)	63.0 (60.0–69.0)	
12–35	66	69.0 (10.6)	65.5 (61.0–75.0)	
36–59	72	74.3 (12.9)	70.5 (65.0–82.0)	
Sex				
Male	150	69.5 (12.0)	65.5 (62.0–73.0)	0.732
Female	107	68.7 (10.3)	67.0 (60.0–74.0)	
Health facility				
ICMH	80	65.9 (10.9)	63.0 (60.0–69.5)	< 0.001
UHC	80	69.5 (9.6)	67.0 (62.0–74.5)	
CC	97	71.6 (12.3)	69.0 (63.0–78.0)	
Child condition				
Calm	122	70.1 (11.2)	67.0 (63.0–74.0)	< 0.001
Asleep	110	67.2 (11.5)	63.0 (60.0–70.0)	
Some movements or crying	25	73.2 (9.5)	72.0 (67.0–78.0)	
RR interpreted by VEP (bpm)				
< 40	154	71.9 (11.8)	69.0 (63.0–77.0)	< 0.001
40–59	76	65.7 (9.9)	63.0 (60.0–68.5)	
≥ 60	27	62.9 (5.9)	62.0 (58.0–68.0)	
Total	257	69.1 (11.3)	66.0 (61.0–73.0)	

*bpm* breaths per minute, *CC* Community clinic, *ICMH* Institute of Child and Mother Health, *IQR* Inter-quartile range, *RR* Respiratory rate, *SD* Standard deviation, *UHC* Upazila Health Complex, *VEP* Video expert panel

**Table 5:** Multiple linear regression to evaluate time to count respiratory rate by ChARM

Factors	Unadjusted coefficient β (95% CI)	***p***-value	Adjusted coefficient β (95% CI)	***p***-value
Age (months)				
< 2	Ref		Ref	
2–11	−2.5 (−7.3, −2.2)	0.296	−3.9 (−8.6, −0.9)	0.109
12–35	0.9 (−4.1, 5.9)	0.734	−1.5 (−7.1, 4.2)	0.609
36–59	6.2 (1.3, 11.1)	0.014	3.4 (−3.1, 9.9)	0.300
Sex				
Male	Ref			
Female	−0.8 (−3.6, 2.0)	0.570		
Health facility				
ICMH	Ref		Ref	
UHC	3.6 (0.1, 7.1)	0.041	−0.3 (−4.1, 3.4)	0.857
CC	5.7 (2.4, 9.0)	0.001	0.5 (−3.4, 4.5)	0.785
Child condition				
Calm	Ref		Ref	
Asleep	−2.9 (−5.8,—0.1)	0.046	1.1 (−2.7, 4.8)	0.581
Some movements or crying	3.1 (−1.7, 8.0)	0.203	7.7 (2.7, 12.6)	0.002
RR interpreted by VEP (bpm)				
< 40	Ref		Ref	
40–59	−6.2 (−9.2, −3.2)	< 0.001	−4.5 (−7.8, −1.1)	0.009
≥ 60	−9.0 (−13.4, −4.6)	< 0.001	−7.1 (−12.2, −1.9)	0.008

*bpm* breaths per minute*, CC* Community clinic, *CI* Confidence interval, *ICMH* Institute of Child and Mother Health, *RR* Respiratory rate, *UHC* Upazila Health Complex, *VEP* Video expert panel

## Discussion

Our study evaluated the performance of the ChARM device to automatically count RR compared to the VEP count among children with suspected pneumonia. The results indicate that the ChARM device is quite reliable in this context, showing high degree of agreement with the VEP.

ChARM and the VEP agreed on RR counts within ± 2 bpm in 68.1% of children, with a mean difference of 1.7 bpm and limits of agreement ranging from −6.7 to 10.2 bpm. Importantly, ChARM demonstrated excellent sensitivity (95.8%) and specificity (93.5%) in classifying fast versus normal breathing. The kappa statistic of 0.86 further underscores the strong agreement between the two methods, suggesting minimal misclassification in identifying fast and normal breathing.

The performance of ChARM in this study was comparable with a study conducted in Ethiopia, where the mean difference between ChARM and the reference standard was −1.1 bpm, wide limits of agreement (−19.6 to 17.4 bpm), and a sensitivity of 81.5% with a kappa of 0.65 [31]. Ghosh et al. evaluated ChARM in children in India and found high inter‑rater agreement, with a kappa value of 0.74 [32].

However, our study found that the agreement in RR count between ChARM and VEP was relatively low in younger children with elevated RR. This observation aligns with Ghosh et al.’s findings, where the least agreement was observed in children aged 0–2 months [32]. In younger children, breathing may be irregular, resulting in difficulty in RR calculation by ChARM. Additionally, higher RR is more prone to misclassification by ChARM, as rapid breathing can lead to overlapping signals and decreased sensitivity in distinguishing individual breaths. Our study also reported poor agreement in measuring and classifying fast breathing when the child was moving or crying. Movement artifacts and crying likely interfere with the ability of an automated device to detect breathing signals. Therefore, to ensure optimal accuracy, RR should ideally be measured when the child is calm and motionless.

Although a high variability in RR counts between ChARM and the VEP was observed, the sensitivity and specificity for the classification of fast breathing were not affected. Only 5.8% of RR counting between ChARM and the VEP fell on opposite sides of the cut-offs for age, which occurred mainly among RR counts on the border of the threshold between normal and fast breathing categories.

This study reported an acceptable performance in terms of time, with a median duration of 66 s required for ChARM to count RR. However, this does not include the time needed to fit the device and allow the child to settle. No previous study has reported the time required by ChARM to generate an RR, as we observed in the present study. The WHO suggests measuring for 60 s to count RR manually to identify fast breathing. A counter that takes a long time to count RR may cause the child to become agitated, which can further lengthen the process and may result in abandoning RR counting altogether in some cases.

The main strength of this study lies in the use of a VEP as the reference standard for evaluating the ChARM device. By systematically assessing the videos and determining the RR, the study effectively mitigates the biases associated with using expert manual RR counts as the reference standard. The VEP has been successfully used in previous studies to count RR and has demonstrated its effectiveness [23, 33]. This methodological approach not only improves the reliability of our findings but also highlights the robustness of the VEP as a tool for precise RR measurement in paediatric populations.

There were some limitations to the study. First, RR could not be measured with the ChARM device in some children who were moving or crying. Additionally, some children were excluded from the analysis because the VEP could not measure RR from videos due to movement or crying during the assessment. While the VEP successfully measured RR in children with some movements or crying (accounting for 9.7% of the analytic sample), the ChARM device demonstrated greater inaccuracy in these cases. Although this proportion was relatively low in our study, this limitation could significantly impact the device’s overall accuracy in clinical settings with higher rates of such conditions. Second, there might be a selection bias in the children who were enrolled, considering that majority of the children with a median age of 13 months, like in our study, were likely to move and sometimes cry when the ChARM device was attached. When the child was uncooperative during the assessment, sufficient time was allowed to calm them before reattempting, and multiple attempts were made to measure RR if necessary. Third, the agreement in RR counts between the ChARM device and VEP was very low in children with elevated RR. Considering that fast breathing is the most sensitive indicator of pneumonia, the ChARM device may have limitations in accurately assessing children who are severely ill. In our study, agreement in RR was defined as a difference of within ± 2, ± 3, ± 4, and ± 5 bpm between the ChARM and the VEP. However, when RR is markedly elevated (e.g., ≥ 60 bpm), these agreement thresholds typically do not affect the classification of breathing as “fast” or “normal”, as such high rates remain distinctly in the “fast” category regardless of minor measurement differences. Fourth, a single ChARM device was used in the project. Therefore, inter-device variation in RR count could not be assessed. Fifth, the presence of the research team and videography procedure might have influenced the child’s RR. The potential impact of attaching this device on the actual RR of the child has not been established in this study. Sixth, the exclusion of some children – particularly those with severe illness or agitation, or whose RR was uninterpretable by the VEP – might introduce bias. These factors may affect the generalizability and reliability of the findings. Seventh, although ChARM measured RR within an acceptable time, the additional time needed for device application and calming children could reduce efficiency in high-pressure or time-sensitive settings.

This study focused on the accuracy of the ChARM device in detecting fast breathing and did not include an analysis of the underlying diagnoses or distinguish between potential causes of fast breathing, such as bronchiolitis, mucus plugs, or reactive airway disease. Furthermore, the study did not differentiate between viral and bacterial causes of fast breathing or assess radiological and laboratory findings to provide a comprehensive diagnostic context. Additionally, the study did not evaluate whether the detection of fast breathing by the device triggered further clinical actions or escalation of care, which limits the understanding of its impact on clinical decision-making. These factors represent significant limitations that warrant further investigation in future studies.

The ChARM device can be highly effective for diagnosing childhood pneumonia in resource-poor settings, where access to trained healthcare workers is limited. Manual RR counting is prone to errors, particularly in understaffed clinics. ChARM can reduce reliance on human expertise, ensure quicker diagnosis and thus support lower-skilled health workers in identifying pneumonia. Its portability and ease of use can make it ideal for low-resource environments. ChARM can improve healthcare delivery and potentially save lives by facilitating prompt pneumonia management. However, the applicability of the device for real world use in busy clinics needs further evaluation, given the time needed for application and the fact that application can trigger crying. Additionally, the affordability of ChARM in low-resource settings should be carefully assessed, as financial constraints often limit the adoption of new medical technologies. Further studies should explore its cost-effectiveness, including initial purchase costs, maintenance expenses, and long-term sustainability in resource-limited healthcare systems.

## Conclusions

Our study demonstrates that while RR counts from ChARM and VEP varied, the performance of ChARM identifying fast breathing was excellent and achieved within an acceptable time. Concerns remain regarding the accuracy of the ChARM device in counting RR for children under two months, those with elevated RR, and those exhibiting movement and crying. Additionally, attaching the device can agitate child, making accurate RR measurement challenging. ChARM can be a viable alternative for RR measurement in children, but only after addressing these issues and conducting further evaluations on a larger sample in real-world settings.

## Trial registration

This study was retrospectively registered with Current Controlled Trials under the identifier ISRCTN14120515 on 19 September 2024.

## Supplementary Information

Supplementary Material 1.

## Data Availability

The anonymized datasets for analysis of the present study are available from the corresponding author on reasonable request.

## Abbreviations

bpm: Breaths per minute
ChARM: Children’s Automated Respiration Monitor
CHCP: Community HealthCare Provider
CI: Confidence Interval
CONSORT: Consolidated Standards of Reporting Trials
ICMH: Institute of Child and Mother Health
IMCI: Integrated Management of Childhood Illness
IQR: Inter-Quartile Range
LMIC: Low- and Middle-Income Country
NPV: Negative predictive value
PPV: Positive Predictive Value
RR: Respiratory Rate
SD: Standard Deviation
UHC: Upazila Health Complex
VEP: Video Expert Panel
WHO: World Health Organization
