# Supplementary material for: Evaluating the performance of an automated respiratory rate counter in detecting fast breathing pneumonia in children using a reference video expert panel
Source: BMC Digit Health. 2025 Aug 21;3(1):32. doi: 10.1186/s44247-025-00175-3 (PMC12442458; doi:10.1186/s44247-025-00175-3)
Supplement: Supplementary file 1 — Supplementary Material 1. [file 44247_2025_175_MOESM1_ESM.docx]

**Supplementary materials**

Supplement to:

Evaluating the performance of an automated respiratory rate counter in detecting fast breathing pneumonia in children using a reference video expert panel

Ahad Mahmud Khan, Md Shafiqul Islam, Nabidul Haque Chowdhury, Salahuddin Ahmed, Rezwana Tabassum, Sadia Afrin, Zannatul Ferdush Amin, Kazi Sazzadul Haque, Afroza Yeasmin Rumi, Jawata Rahman, Rakib Bhuiyan, Rizouan Ur Rashid, Kamrun Nahar, Robynne Simpson, Ayaz Ahmed, Md Mozibur Rahman, Ting Shi, Abdullah H Baqui, Steve Cunningham, Eric D McCollum, Harry Campbell

**Corresponding author:**

Ahad Mahmud Khan

Projahnmo Research Foundation, Dhaka, Bangladesh

Email: ahad_mahmud@hotmail.com

**Supplementary figure 1: Respiratory rate interpretation from video recording by video expert panel**


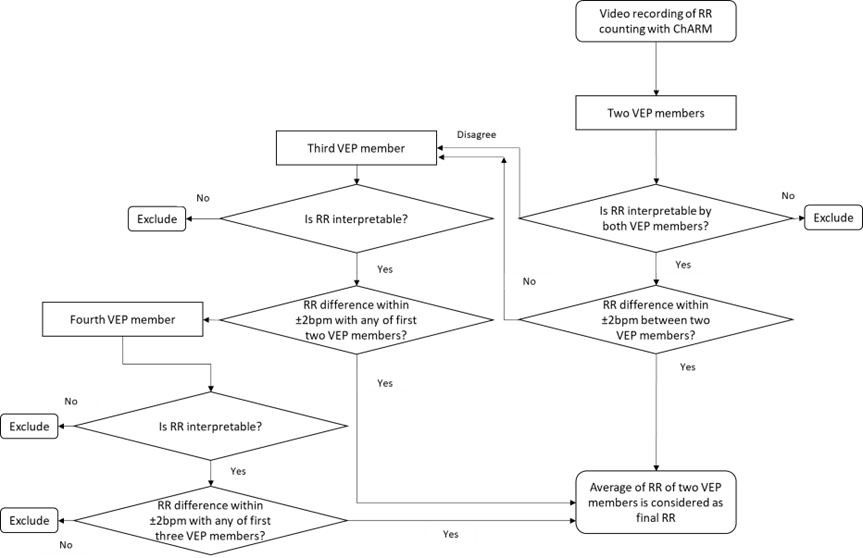


**Supplementary figure 2: Bland-Altman plot showing the agreement between the respiratory rate count measured by the ChARM and the respiratory rate count assessed by the video expert panel**


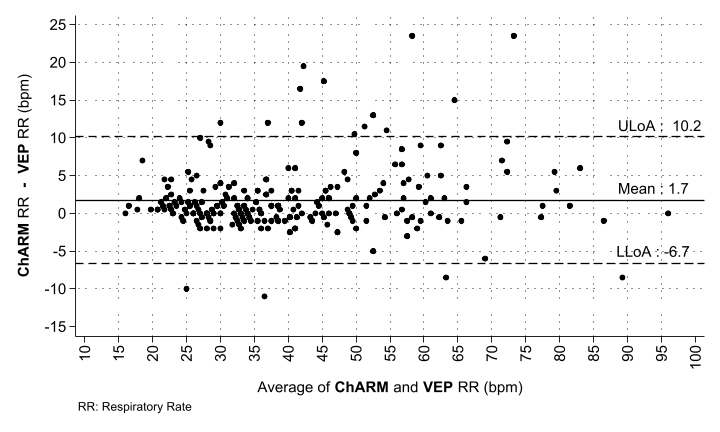


Supplementary Table 1: Accuracy of the ChARM in identifying fast breathing compared to video expert panel

| **Characteristics** | **Total number** | **Fast breathing N (%)** | **Sensitivity (%)** | **Specificity (%)** | **PPV (%)** | **NPV (%)** | **Agreement (%)** | **Kappa** |
| --- | --- | --- | --- | --- | --- | --- | --- | --- |
| **Age (months)** | | | | | | | | |
| 0-2 | 25 | 9 (36.0) | 88.9 | 100.0 | 100.0 | 94.1 | 96.0 | 0.91 |
| 2-11 | 94 | 33 (35.1) | 100.0 | 86.9 | 80.5 | 100.0 | 91.5 | 0.82 |
| 12-35 | 66 | 24 (36.4) | 91.7 | 95.2 | 91.7 | 95.2 | 93.9 | 0.87 |
| 36-59 | 72 | 5 (6.9) | 100.0 | 97.0 | 71.4 | 100.0 | 97.2 | 0.82 |
| **Sex** | | | | | | | | |
| Male | 150 | 34 (22.7) | 94.1 | 93.1 | 80.0 | 98.2 | 93.3 | 0.82 |
| Female | 107 | 37 (34.6) | 97.3 | 94.3 | 90.0 | 98.5 | 95.3 | 0.90 |
| **Health facility** | | | | | | | | |
| ICMH | 80 | 34 (42.5) | 97.1 | 91.3 | 89.2 | 97.7 | 93.8 | 0.87 |
| UHC | 80 | 22 (27.5) | 100.0 | 87.9 | 75.9 | 100.0 | 91.2 | 0.80 |
| CC | 97 | 15 (15.5) | 86.7 | 98.8 | 92.9 | 97.6 | 96.9 | 0.88 |
| **Child condition** | | | | | | | | |
| Calm | 122 | 34 (27.9) | 94.1 | 96.6 | 91.4 | 97.7 | 95.9 | 0.90 |
| Asleep | 110 | 28 (25.5) | 96.4 | 96.3 | 90.0 | 98.8 | 96.4 | 0.91 |
| Moving or crying | 25 | 9 (36.0) | 100.0 | 62.5 | 60.0 | 100.0 | 76.0 | 0.55 |
| **Total** | **257** | **71 (27.6)** | **95.8** | **93.5** | **85.0** | **98.3** | **92.2** | **0.86** |

CC – Community clinic, ICMH – Institute of Child and Mother Health, NPV – Negative predictive value, PPV – Positive predictive value, UHC – Upazila Health Complex, VEP – Video expert panel
